# Health Impact of PM_10_, PM_2.5_ and Black Carbon Exposure Due to Different Source Sectors in Stockholm, Gothenburg and Umea, Sweden

**DOI:** 10.3390/ijerph14070742

**Published:** 2017-07-07

**Authors:** David Segersson, Kristina Eneroth, Lars Gidhagen, Christer Johansson, Gunnar Omstedt, Anders Engström Nylén, Bertil Forsberg

**Affiliations:** 1 Swedish Meteorological and Hydrological Institute, 60176 Norrköping, Sweden; lars.gidhagen@smhi.se (L.G.); gunnar.omstedt@smhi.se (G.O.); 2 Environment and Health Administration, 38024 Stockholm, Sweden; kristina.eneroth@slb.nu (K.E.); christer.johansson@aces.su.se (C.J.); anders.engstrom.nylen@slb.nu (A.E.N.); 3 Environmental Science and Analytical Chemistry, Stockholm University, 11418 Stockholm, Sweden; 4 Occupational and Environmental Medicine, Umea University, 90187 Umeå, Sweden; bertil.forsberg@umu.se

**Keywords:** dispersion modeling, exposure, particulate matter, residential wood combustion, health impact assessment

## Abstract

The most important anthropogenic sources of primary particulate matter (PM) in ambient air in Europe are exhaust and non-exhaust emissions from road traffic and combustion of solid biomass. There is convincing evidence that PM, almost regardless of source, has detrimental health effects. An important issue in health impact assessments is what metric, indicator and exposure-response function to use for different types of PM. The aim of this study is to describe sectorial contributions to PM exposure and related premature mortality for three Swedish cities: Gothenburg, Stockholm and Umea. Exposure is calculated with high spatial resolution using atmospheric dispersion models. Attributed premature mortality is calculated separately for the main local sources and the contribution from long-range transport (LRT), applying different relative risks. In general, the main part of the exposure is due to LRT, while for black carbon, the local sources are equally or more important. The major part of the premature deaths is in our assessment related to local emissions, with road traffic and residential wood combustion having the largest impact. This emphasizes the importance to resolve within-city concentration gradients when assessing exposure. It also implies that control actions on local PM emissions have a strong potential in abatement strategies.

## 1. Introduction

Health impact assessments of different air pollution source contributions are important for finding the most effective abatement measures. Air quality dispersion modeling of population exposures has been used in studies to assess effects on the global population (e.g., [1,2]), the European population (e.g., [3,4,5]), as well as for specific countries (e.g., [4,6,7,8,9]) and urban areas (e.g., [7,10,11]). In many studies, the contribution to the exposure from specific anthropogenic sources is separated in order to assess health impacts of abatement strategies (e.g., [3,4,11,12,13,14]).

Most commonly PM_2.5_ is used as indicator to estimate effects on the mortality, as recommended for cost-benefit analysis by the World Health Organization (WHO) in the HRAPIE project [15]. Ambient PM_2.5_ was the fifth-ranking mortality risk factor in 2015. Exposure to PM_2.5_ caused 4.2 million deaths globally (95% uncertainty interval, 3.7–4.8 million) [2]. Other PM metrics have also been used, such as PM_10_ [6,9], elemental carbon (EC) and black carbon (BC) [7,16].

As pointed out by the WHO [17], there is convincing evidence that PM, almost regardless of source, has detrimental health effects. An exception is sea salt, for which there is little indication of harmfulness [17]. There is however more evidence of health effects linked to combustion sources than non-combustion sources. In fact, an important argument for using BC instead of PM_2.5_ in health impact assessments of local emissions, is that BC is a more specific indicator of the local combustion particles. This is due to PM emitted from vehicle combustion containing a large fraction of BC in comparison with other large source categories. Recent epidemiological studies indicate that associations between adverse health effects and exposure are stronger for BC than for PM_2.5_ [18,19]. In addition to having significant health impacts, BC is also an important short-lived climate forcer [20].

The most important anthropogenic sources of primary PM in Europe are exhaust and non-exhaust emissions from road traffic and combustion of solid biomass [21], but the relative importance of these varies drastically between cities. Local contributions to PM_2.5_ include the combustion generated PM together with mechanically generated particles from wear of brakes, tires and road surfaces, as well as other suspended non-combustion material [22] and this is of particular importance in Nordic cities [23,24,25,26]. Another important source to PM_2.5_ in Europe is residential biomass combustion, for which emissions, in contrast to most other PM_2.5_ sources, are increasing [27]. Secondary PM also contributes significantly, for example due to combined emissions of NO_x_, NH_3_ and SO_2_ in densely populated parts of Europe and from biogenic VOC emissions in northern Europe [28,29].

An important issue in health impact assessments is what metric, indicator and exposure-response function to use for different types of PM. Historically many epidemiological studies have used measurements from a central-site monitoring station to estimate exposure. Although temporal variability may be reasonably captured, the spatial variability—the within-city gradients—will be underestimated [30]. Describing the spatial variations in concentrations becomes particularly important when dominating local sources are located in the direct vicinity of the exposed population. In order to reduce the risk of exposure misclassification, methods with higher spatial resolution, such as Land Use Regression (LUR) models and meteorological dispersion models have recently become increasingly popular [31]. There are benefits and disadvantages related to both methods; while LUR requires a large investment in monitoring, dispersion modeling requires a detailed emission inventory. An advantage of using dispersion modelling is that it allows apportionment of different local sources.

The objective of this study has been to estimate and evaluate exposure to respirable PM from different sources with appropriate temporal and spatial resolution for use in epidemiological studies and health impact assessments. Different indicators for PM exposure and different exposure-response functions for different source categories are applied in a novel way and the consequences when performing health impact analysis are investigated.

## 2. Materials and Methods

Given the availability of detailed emission inventories for the three major cities included in this study: Stockholm, Gothenburg and Umea, we have applied dispersion modeling to assess annual mean exposure to PM_2.5_, PM_10_ and BC. Impact assessments and exposure calculations for the three cities were performed for year 2011. The modeling domains were selected to cover the areas with population-based cohorts available for epidemiological studies. The sizes of the model domains were 92.8 × 112 km^2^ (Gothenburg), 174 × 236 km^2^ (Stockholm) and 108.8 × 182.4 km^2^ (Umea), covering the city cores with suburbs, but also a large number of smaller villages and some rural areas. However, the exposure and impact assessment presented here is limited to a square of 35 × 35 km^2^, centered on the main city of each domain. Efforts were made to ensure that the methods applied in the three cities were as similar as possible, allowing comparison of the results.

**Figure 1:**
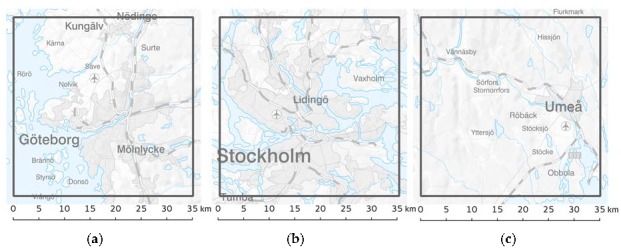
The assessment was limited to squares of 35 × 35 km^2^ around each of the three cities (**a**) Gothenburg (**b**) Stockholm and (**c**) Umea. Reproduced from [32].

The assessment areas are presented in Figure 1. Stockholm is the largest of the three cities, with a population of about 1.7 million, followed by Gothenburg with a population of almost 700,000 and Umea with 110,000 people living inside the 35 × 35 km^2^ areas. Both Stockholm and Gothenburg are metropolitan areas, while the Umea area is a medium sized Swedish city.

### 2.1. Emission Inventories

The following source categories were defined in order to allow source apportionment and separate impacts of the main contributors to particle emissions in urban areas in Sweden:

- road traffic exhaust
- road traffic non-exhaust
- RWC (residential wood combustion)
- shipping
- other (mainly industrial processes, energy production, off-road machinery, agriculture)

**Figure A1:**
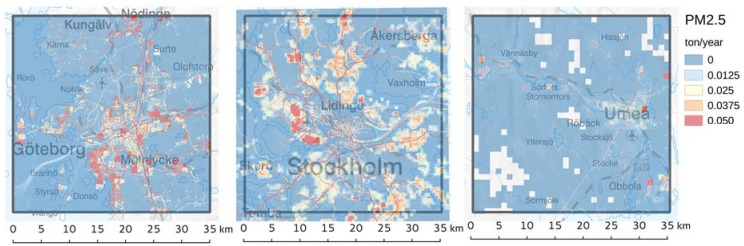
Emissions of PM_2.5_ during year 2011. All source categories are included. Point sources and line sources are gridded with a spatial resolution of 100 × 100 m^2^. Reproduced from [32].

For all three cities, there were local or regional bottom-up emission inventories available. The emission database for the county of Stockholm, administered by the Air Quality Management Association of Eastern Sweden, is updated annually and contains detailed information about emissions from e.g., road and ferry traffic, petrol stations, industrial areas and domestic heating [33]. For Gothenburg, the available emission inventory was provided by the city’s environmental administration and for Umea, a detailed inventory was available from the national dispersion modeling system SIMAIR (Swedish Meteorological and Hydrological Institute, Norrköping, Sweden) [34]. All emission databases were compiled using the Airviro air quality management system (Swedish Meteorological and Hydrological Institute and Apertum IT AB, Norrköping, Sweden) [35], making them similar in structure. As an example, the spatial distribution of emissions of PM_2.5_ is presented in Appendix A, Figure A1. Data files for all PM-fractions are also provided as Supplementary Materials, S1.

Although the exposure assessment is presented for the areas specified in Figure 1, the emission inventories and the modeling domains had an extent more than 10 km larger than the assessment areas, ensuring that impact contributions from emission sources just outside of the assessment area were also included.

#### 2.1.1. Road Traffic

For all three cities, the road networks were described with a high level of detail. Measured traffic flows, separating heavy and light duty vehicles, were available for most major roads and elsewhere completed with modeled traffic flow [34].

The vehicle fleet composition was derived from the national vehicle registry. Vehicles are grouped into passenger cars (petrol, diesel, ethanol and gas), light commercial vehicles (petrol and diesel), heavy goods vehicles (petrol and diesel) and buses (diesel, gas and ethanol). Emission factors for PM-exhaust for different vehicle types, speeds and driving conditions are calculated based on HBEFA 3.1 [36]. BC emission factors are based on the TRANSPHORM project [37], but corrected based on local measurements at a street canyon site in Stockholm [38].

Non-exhaust emissions include road wear and some contributions from brake wear and tire wear. A relation between exhaust and non-exhaust emissions of PM_10_ has been estimated from measurements in a street canyon and using NO_x_ as a tracer [39]. For Stockholm, the ratio of non-exhaust to exhaust PM_10_ emissions is on average a factor 12 depending on speed and road type, while for Gothenburg and Umea it is 8. The higher ratio obtained for Stockholm relates to differences in the share of studded tires as well as meteorological conditions (amount of precipitation and time of wet-, snow- or ice-covered roads). The former is important for road wear and the latter for the road wear and the suspension of dust [24,25].

A similar approach is applied for PM_2.5_, where the total PM_2.5_ emission from road traffic is estimated as *PM_exhaust_ + A* × *(PM_10-from-road-traffic_* − *PM_exhaust_)*, where the factor *A* is estimated from measurements. For Stockholm the factor *A* was 0.3, while for Gothenburg and Umea, a value of 0.2 was used. This means that we assume that 20–30% of the non-exhaust PM less than 10 µm is PM_2.5_, which is rather consistent with the expected share of PM_2.5_ of the emission factors for wear of brakes, tires and roads.

#### 2.1.2. Residential Heating

The source category “residential heating” includes emissions from stoves and boilers (typically <60 kW) in single-family houses as well as stoves used in apartments and small boilers used for heating multi-family houses. Around 98% of the emissions of PM from residential heating are caused by combustion of solid fuels (wood logs or pellets). Emission factors for PM_10_ and PM_2.5_ are based on Omstedt et al. [40] and the fraction of PM_2.5_ corresponding to BC is taken from EMEP/EEA Inventory Guidebook 2013 [41].

In Västerbotten County, including the Umea area, a database has been constructed by collecting register data from chimney sweepers. This meant that a detailed inventory of individual stoves and boilers was available, allowing most of the emissions from residential heating to be represented as point-sources [40]. This database has also been used to conduct interviews about domestic wood burning. A total of 10,287 appliances were represented as point sources within the assessment area. A minor fraction of the appliances had not been geo-referenced and their emissions were instead distributed spatially using gridded proxy data, such as number of appliances (stoves, boilers) per municipality, living space of small houses per km^2^, population density per 100 × 100 m^2^ and availability of district heating. Emissions from boilers were reduced in areas with district heating, while emissions from stoves, which are mainly used for complementary heating and comfort, were distributed without reduction in all residential areas. In Stockholm and Gothenburg, there was no bottom-up inventory of residential appliances available. For this reason, all emissions from residential heating in these areas were gridded using a similar methodology. The gridded emissions were represented with a resolution of 100 × 100 m^2^ and a height of 2 m in the dispersion model, i.e., the release height and plume lifts of different individual appliances are not considered. The energy consumption for the residential heating in Stockholm and Gothenburg was obtained from energy balance calculations by Statistics Sweden (the national statistics agency). For Umea, the detailed inventory of stoves and boilers also included a survey on firing habits and wood consumption that was used to estimate the energy consumption.

#### 2.1.3. Ship Emissions

Emissions from shipping were described using a bottom-up approach including actual ship movements of all ships equipped with Automatic Identification System (AIS) transponders and ship properties acquired from international databases. The calculations are similar to those described by Jalkanen et al. [42]. Since only yearly average concentrations was of interest, the annual average distribution of emissions was used in the modeling and introduced as grids with a resolution of 1 × 1 km^2^.

#### 2.1.4. Other Sources

The source category “other” consists of emissions from energy production, industrial processes, off-road machinery and agriculture. Emissions from off-road machinery and agriculture were taken from the yearly Swedish compilation of gridded emissions to air, in which the national total emissions are distributed spatially with a resolution of 1 × 1 km^2^, using statistics and spatial proxies [43]. Examples of statistics used are number of take-offs at airports or total power of tractors registered for a specific municipality. Examples of spatial proxies are harbor areas, industrial areas, airports, agricultural fields. Large point-sources were represented individually in the inventories with actual coordinates. For Stockholm emissions from off-road machinery were disregarded due to their diffuse nature and unknown temporal and spatial variability. PM and BC emissions from industrial processes were also disregarded in Stockholm due to their low importance compared to the other sources. This means the “other” source category for Stockholm mainly consists of stationary combustion.

### 2.2. Model Simulations

**Figure 2:**
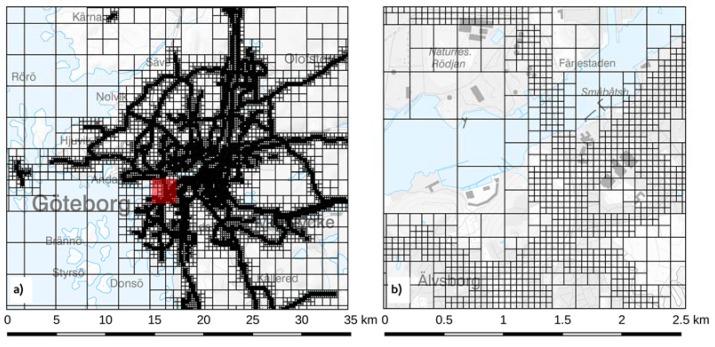
The quadtree receptor grid applied for dispersion modelling of road traffic in Gothenburg. (**a**) the grid of the whole assessment area. (**b**) a close-up of an area in central Gothenburg, showing the receptor grid of 50 × 50 m^2^ close to major roads and a coarser grid further away. The location of the close-up is shown in (**a**) as a red square. Reproduced from [32].

Gaussian models included in the Airviro air quality management system have been used to simulate the annual average PM contributions from different local sources during the year 2011 [35,44]. The LRT contributions to each domain were taken from measurements at rural sites outside the calculation domains or determined indirectly (see below). To reduce the time for computations while still maintaining a high resolution in the vicinity of roads and point sources, a quadtree [45] receptor grid was used. The receptor grids had an original coarse resolution ranging from around 3 × 3 km^2^ in rural areas without any emission sources and successively down to a minimum of around 35 × 35 m^2^ along major roads and close to stacks. The receptor grid used for road traffic in Gothenburg is presented as an example in Figure 2. The spatial resolution of the receptor grid used for the different modeling domains and source categories is given in Table 1.

Airviro makes use of a diagnostic wind model based on the concept first described by Danard [46], in which it is assumed that small scale winds can be seen as a local adaptation of large scale winds. For Gothenburg and Umea, dispersion modelling was performed for each hour of the year, while in Stockholm meteorological conditions were based on a climatology (a representative sample) created from 15 years of meteorological measurements.

The meteorological data was grouped into 60 wind direction classes with six stability classes within each wind sector. Concentration fields were calculated for each of the classes and weighted according to their frequency of appearance in the climatology [44]. The reason for not performing hourly calculations in Stockholm was to reduce the computational time. To ensure consistency in annual averages estimated using the two methods, a comparison was made between annual mean values for 7000 receptor points in the Greater Stockholm area. The comparison shows that the results are very similar; slope = 0.891 ± 0.002; intercept = 0.071 ± 0.021; correlation, r = 0.99, indicating that the climatology based calculations well represents the full hourly time-series.

For Gothenburg and Umea a slightly different Gaussian model was applied, using a finite length line source model for road sources as described and evaluated by Omstedt et al. [47] and further evaluated by Gidhagen et al. [48]. For point sources in Umea and Gothenburg, the OML (Operationelle Meteorologiske Luftkvalitetsmodeller, Operational Meteorological Air Quality Models) point-source model was applied [47,49].

Contribution from RWC was described using gridded emission data in Gothenburg and Stockholm and by individual point-sources for Umea. To evaluate the influence this might have on the calculated exposure, a comparison was made (see Appendix B). The comparison shows high correlation and good agreement on average, but indicates that using gridded emission might cause an overestimation of up to 25% in areas with the highest concentrations and a slight underestimation in the areas with lowest concentrations.

To increase the amount of data available for comparison with measurements, modelling was also performed for year 2000 and in the case of Stockholm 2005, following the same methodology. Year to year variations of the emissions were assumed to be small, allowing interpolation of the annual average contributions from local sources for the years 2000–2011.

### 2.3. Determination of Long-Range PM Contributions

The method chosen to quantify the LRT contribution was by identifying a background station, either from a rural area outside the modeling domain or inside the modelling domain. This means that no source attribution is made for LRT. It also means that secondary PM, which is mainly generated on time-scales larger than the residence times within the modelling domain, is included in the LRT contribution. Since the required type of background monitoring data is sparse, sometimes not available for a particular year and normally only for PM_10_ or PM_2.5_, it was necessary to use different approaches for the three modeling domains.

For Stockholm, annual mean LRT contributions of PM_10_ and PM_2.5_ concentrations were taken from the rural background station Norra Malma, located some 60 km northeast of Stockholm. Measured mean concentration for BC was taken from the rural station Aspvreten, situated 80 km south of Stockholm. These levels (presented in Table A1), were added to the calculated local contributions of the Stockholm domain.

For Gothenburg, an urban background station, Femman, has been collecting PM_10_ levels at roof level in the city center since 1990, using a TEOM (Tapered Element Oscillating Microbalance), model 1400A, Rupprecht & Patashnick Inc., Albany, NY, USA). By summing up annual average contributions from all simulated local sources and then subtracting the sum from the measured annual average, a rest attributed to long-range transport remains. This indirect estimation of LRT means that results are adjusted to match measurements at Femman. This ensures a good exposure estimate in central parts of Gothenburg, but also means that uncertainties in the description of local sources will affect the calculated LRT. For 2011 the measured total PM_10_ concentration was 17.7 µg·m^−3^, the local model contribution was 7.1 µg·m^−3^ and thus the long-range contribution was estimated to 10.6 µg·m^−3^. PM_2.5_ was not measured at Femman during 2011, but from parallel measurements during the years prior to 2011 the PM_2.5_/PM_10_ ratio for 2011 could be estimated to 0.42, yielding 7.4 µg·m^−3^ at Femman. With a model contribution from local sources of 3.3 µg·m^−3^, the LRT contribution for PM_2.5_ was estimated to 4.1 µg·m^−3^. The ratio PM_2.5_/PM_10_ of 0.42 for 2011 in Gothenburg is relatively low compared to measurements in other parts of Sweden. This may be caused by uncertainties in available measurements, but it could also indicate that there are local sources that have not been accounted for in the dispersion modeling. There is however not sufficient data available to draw any conclusions regarding this. For BC there are no historical measurements from the area. Instead BS (black smoke) data, collected at a rural station outside Gothenburg, was converted to BC as measured with a transmission method, using a relation from urban conditions in Stockholm [50]. The annual average long-range contribution of BC was estimated to 0.2 µg·m^−3^.

For Umea, a rural background station, Vindeln, is located 50 km northwest of Umea, at which PM_10_ was measured from 2002 to 2008. Output of secondary inorganic aerosols (SIA) from a European scale model assessment 1990–2013 [51], using a regional dispersion model MATCH [52], showed a good correlation with measured PM_10_ at Vindeln. Applying an expression (SIA2011) + 3.4 µg·m^−3^ the annual averaged PM_10_ at Vindeln for 2011 was estimated to 6.8 µg·m^−3^. From a three year record of parallel PM_2.5_ and PM_10_ measurements at the rural station Bredkälen, located 245 km west of Umea and outside the modeling domain, the ratio PM_2.5_/PM_10_ in long range transport was estimated to 0.56. Assuming this ratio to be valid also at Vindeln, the annual PM_2.5_ level for 2011 was estimated to 3.8 µg·m^−3^. For BC there is no reliable information. Some BS data exists from smaller cities in the modeling domain, but only up to 2002, ending on 8–9 µg·m^−3^. However, these data are affected by local wood combustion and are not representative for the long range transported fraction. Based on the assumption of a south-north gradient in BC levels over Scandinavia, we have used the Stockholm value (0.3 µg·m^−3^) to estimate an annual average concentration of BC in Umea of 0.2 µg·m^−3^.

### 2.4. Exposure Contributions and Health Impact Calculations

Exposure has been calculated for population data on a 100 × 100 m^2^ grid acquired from Statistics Sweden, representing the year 2012. The population data is based on the coordinates for the home addresses of the total population, but also includes age separated classes: 0–1 years, from 1–5 years and every five year interval up to 85 years, then a final class >85 years. Both the population data and the calculated concentrations were resampled to a grid with resolution 50 × 50 m^2^ in order to calculate the population weighted concentrations. Since grids were aligned and the same map projection was used, no interpolation was necessary when resampling. Baseline mortality representing all natural deaths was acquired from the Swedish Cause of Death Register at The National Board of Health and Welfare. To avoid overestimation, premature deaths due to the pollutant exposure were subtracted from the baseline. Population and baseline mortality for the three modeling domains are presented in Table 2.

Exposure-response relationships from literature were applied to estimate premature mortality. The analysis was made separately for the population exposure related to the different source categories. To avoid double counting, only one particle fraction was used to represent the impact of each source category in the health assessment. Road wear is the sole contributor to locally emitted coarse particles (PM_2.5–10_) that is taken into account. In Sweden, the emissions from vehicle generated road wear and re-suspension of dust are large, mainly due to the extensive use of studded tires during winter. A relative risk factor for this particular fraction, considered representative for Swedish conditions, was acquired from Meister et al. [53]. The health impact from the long-range contribution is represented by PM_2.5_, using the relative risk factor recommended by WHO and after subtracting 2 µg·m^−3^, considered the lowest exposure level in significant associations [54]. The relative risk factors recommended by WHO are based on studies where differences between cities have been used to calculate the related risks. This is considered well suited to represent LRT, which is not expected to vary significantly within the assessment area.

For particles originating from local sources, three different exposure-relationships are used to demonstrate the range of the estimates. Jerrett et al. [55], investigated associations within a metropolitan area (Los Angeles), providing relative risk factors for PM_2.5_. Hoek et al. [54] combined a lot of studies, mostly “between-city comparisons” and also provide risk factors for PM_2.5_. Janssen et al. [18] provide an estimate for BC and Hoek et al. [54] for EC, both actually represent a mixture of BC, EC and BS measurements and report the same most probable value for the relative risk (60% per 10 µg·m^−3^), but slightly different confidence intervals. In Hoek et al. [54] many of the studies that use EC as an indicator also use a fine spatial resolution (address level). Here the reference by Janssen is used, since the meta estimates were almost identical. Since gradients within the assessment areas are resolved in this study, the relative risk factors that are applied should also be based on within-city comparisons. This should be considered when comparing the results from the three methods. A European multi-cohort study from the ESCAPE Project [56] with associations resulting from “within-city contrasts” in PM_2.5_, found an estimate closer to the one from Jerrett et al. Using different relative risk factors for LRT than for local sources can be further justified by the large differences in the composition of PM. While LRT represents an aged aerosol with more secondary PM, the locally generated PM in urban areas often contains a larger portion of “fresh” combustion particles. In our health impact assessment, factors for local PM_2.5_ represent both combustion and fine wear particles. The relative risks are presented in Table 3.

The applied relative risk factors for BC and PM_2.5_ are taken as representative for the population above an age of 30 years. Consequently, the presented concentrations have been weighted using the same age threshold. For coarse particles, mortality in all ages was used by Meister et al. [53] and the whole population is therefore used in the calculations.

### 2.5. Model Evaluation

All available concentration measurements in the three cities of PM_10_, PM_2.5_ and BC from the period 2000–2011 have been collected for evaluation of the results. When comparing to traffic stations located in street canyons, an additional contribution has been simulated with the OSPM model [57]. Stations used for LRT determination have been excluded from the evaluation.

For Stockholm we have used data from three curbside (traffic) monitoring sites and one urban background site. All three traffic sites: Hornsgatan, Sveavägen and Norrlandsgatan are located in street canyons (i.e., with multistoried buildings on both sides of the street). Traffic intensities and street geometries are different for the different sites. The urban background site, Torkel Knutssonsgatan, is located in central Stockholm on the top of a building 24 m above the street. More detailed descriptions of the sites in Stockholm are provided in Krecl et al. [58] and Targino et al. [59]. PM_10_ and PM_2.5_ was monitored using a TEOM 1400A (Rupprecht & Patashnick Inc., Albany, NY, USA). BC was measured using aethalometers (AE33 and AE31, Magee Scientific, Berkeley, CA, USA) and custom built PSAPs [60].

In Gothenburg, excluding the Femman station, there were only two more stations with measurements of PM_10_ and PM_2.5_, both being traffic stations located along major roads. The station Gårda in Gothenburg is located close to a four-lane motorway running in north-south direction. East of the station there is a hill with small houses and to the west there are irregular multistoried buildings. The Haga station is located in a one-sided street canyon in central Gothenburg with a park to the east of the station. At both stations PM_2.5_ and PM_10_ was monitored using a TEOM 1400AB (Thermo Fisher Scientific, Waltham, MA, USA).

Two stations were available for Umea, an urban background station located at roof of a building in the city center (named Biblioteket) and a curbside traffic station located in a street canyon (named Västra Esplanaden). Both PM_10_ and PM_2.5_ was monitored using a TEOM 1400A (Thermo Fisher Scientific, Waltham, MA, USA).

## 3. Results

### 3.1. Model Evaluation

**Figure 3:**
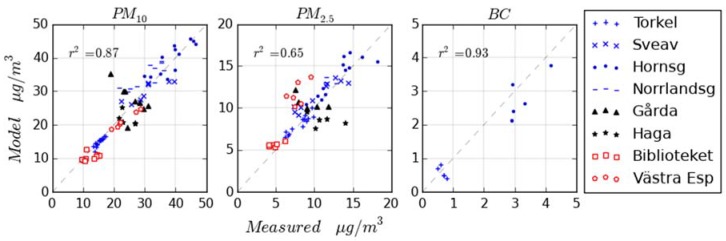
Comparisons between measured and modeled annual average concentrations. The dashed lines are 1:1 lines. Markers representing monitoring stations are blue for Stockholm, red for Umea and black for Gothenburg. PM: particulate matter.

In Figure 3, the comparison between measured and modeled annual average concentrations is presented. The monitoring stations included in the evaluation are described in chapter 2.5 (the names are shortened to fit in the legend). For Gothenburg, there are only curbside stations available for the evaluations (the urban background station Femman was used to estimate LRT and can therefore not be used in the evaluation). For BC, there is only data available for Stockholm. Modelled and measured average concentrations agree well at most monitoring stations, but the trend over the years 2000–2011 is not captured at some sites, especially in Gothenburg (black markers). This is probably due to changes, e.g., of traffic flow, at these sites that have not been described correctly in the modelling. A more detailed statistical comparison is given in Appendix C (see Table A1 for PM_10_, Table A2 for PM_2.5_ and Table A3 for BC). Observational data used in the evaluation is also provided as Supplementary Materials, S3.

### 3.2. Characteristics of PM Exposure and Sectorial Contributions

**Figure 4:**
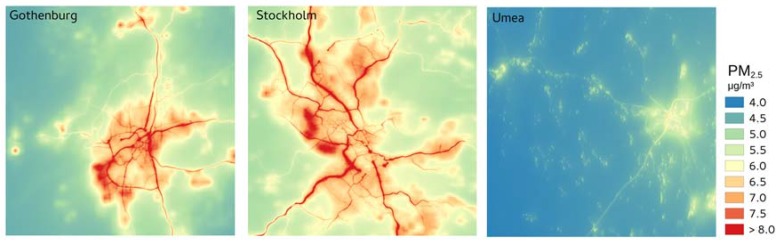
Modeled annual average concentration of PM_2.5_ for year 2011 presented for the three assessment areas of 35 × 35 km^2^.

Figure 4 presents maps of modeled annually averaged PM_2.5_ concentrations. The spatial patterns are dominated by gradients in the concentration fields along major roads and by elevated concentrations in residential areas with small scale combustion. Patterns are similar for PM_10_ and BC. Data files for all concentration fields are included as Supplementary Materials, S2.

**Figure 5:**
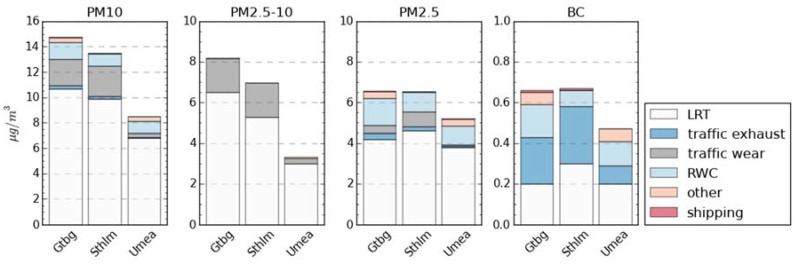
Population weighted exposure (population >30 years) for the three cities and for the selected sectors. For traffic wear, the whole population is used for consistency with calculations of premature mortality. BC: black carbon; LRT: long-range transport; RWC: residential wood combustion.

Figure 5 shows population weighted annual average source contributions to PM_10_, PM_2.5–10_, PM_2.5_ and BC (also in Table A4). It can be seen that the coarse mode (PM_2.5–10_) is mainly from LRT and traffic wear particles. For finer particles (PM_2.5_), the contribution from traffic wear is smaller and emissions from RWC give a significant contribution. The contribution from shipping is small for all the evaluated PM-fractions and within all three assessment areas. For BC, traffic exhaust emissions and RWC cause the largest local contribution. It should be remembered that the emissions from machinery are not included in the “other” source category for Stockholm. In Table 4 the percentage of population weighted concentrations related to local sources is presented.

### 3.3. Health Impact Assessment

**Figure 6:**
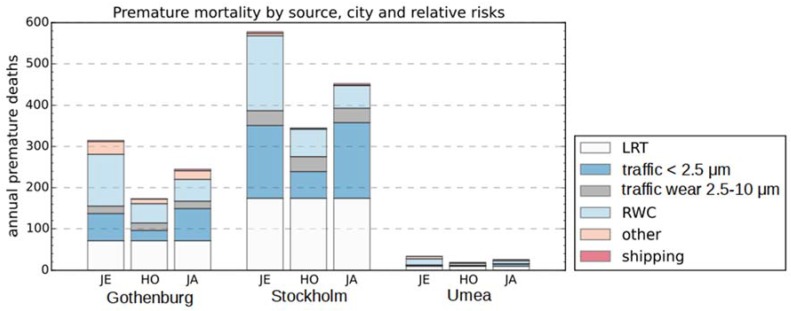
Premature deaths estimated for the different modeling domains. Estimates have been made separately for the source categories. For premature deaths due to local contributions of PM_2.5_ and BC, estimates have been made using relative risks from three different studies: JE: Jerrett et al. [55], HO: Hoek et al. [54], JA: Janssen et al. [18].

Premature mortality for each of the different source categories is presented in Figure 6 (also provided in Table A5). Since relative risks for the local contribution of PM_2.5_ from road traffic represent both combustion and fine wear particles, the sector is here named “traffic < 2.5 µm” instead of “traffic exhaust” and “traffic wear” is referred to as “traffic wear 2.5–10 µm”. For BC, the local contribution is strictly limited to combustion sources. The results indicate that RWC and road traffic are responsible for the majority of the premature deaths. Using BC instead of PM_2.5_ as an indicator, with relative risk factors according to Janssen et al. [18], the relative impact of traffic exhaust increases significantly. For Stockholm, the percentage of the premature deaths caused by local sources that are related to “traffic < 2.5 µm” is estimated to 66% when using BC as an indicator and around 40% using PM_2.5_. In Umea RWC represents 40–60% of the premature deaths due to local sources. For Gothenburg and Stockholm, RWC is somewhat less important and represents 20–50% of the premature deaths related to local sources. In Table 5, the percentage of premature deaths that are attributed to local sources is presented. Using Jerret et al. [55], representing within-city gradients of PM_2.5_, and Janssen et al. [18] representing “between-city gradients” of BC, the local sources represent ≥60% of the premature deaths. Using Hoek et al. [54], LRT and local sources each represent near half of the premature deaths.

## 4. Discussion

Probably the most important source of uncertainty in the calculated concentrations originates in the emission inventories. However, there is not enough information available to perform a quantitative uncertainty analysis. To provide an indication on the uncertainty, ranges are provided based on judgment of the authors. RWC is one of the largest and also one of the most uncertain emission categories, especially for Gothenburg and Stockholm, where no detailed inventory of stoves and boilers is available. Considering uncertainties in fuel consumption, incomplete information regarding technology and their emission factors, the uncertainty for overall emissions from RWC may be at least 50%. For road traffic the uncertainty is lower, but could still be as high as 25% and higher for BC. Emissions from shipping and other sources also have significant uncertainties in the range of 25–50%, but have a smaller influence on the total concentration.

Considering that most of the uncertainty in local contributions is probably related to uncertainties in the emission inventories, relatively simple Gaussian models have been chosen for the dispersion modelling. An advantage of the Gaussian models applied is their computational efficiency, which makes it possible to use a receptor grid fine enough to resolve the strong concentration gradients in the vicinity of roads and close to other sources. For homes close to major roads, the concentration can often be twice as high as the urban background concentration, even without considering street-canyon effects. Capturing these spatial variations is expected to increase the accuracy of the exposure calculated at homes. For annual averages of concentrations, uncertainties in the size of the emissions are transferred proportionally. However, uncertainties in the location of emission sources, which can be significant for RWC emissions in Gothenburg and Stockholm, have a non-linear relation to uncertainties in the concentrations. Further research is needed to quantify the sensitivity of modelled concentrations due to uncertainties in the spatial representation of the emissions.

The comparison between model results and measurements verifies that the calculated total concentration is well captured with the applied methodology. It is however more challenging to evaluate the relative contributions of the different source categories. The results indicate that RWC is generally the dominating source behind exposure to combustion particles. With this in mind it is of interest to compare with source assessments based on other methods. Source assessments of the soot aerosol in Gothenburg using a radio-carbon methodology have been reported by Szidat et al. [61]. They estimated the fraction of EC related to wood combustion at the urban background station Femman in Gothenburg to be 10 ± 2% both during three winter weeks of 2005 and during three summer weeks of 2006. Similar measurements performed at the urban background station Torkel Knutssonsgatan in central Stockholm [62] shows a large variation in the fraction of SC (“soot carbon”) related to combustion of biogenic fuels. Measurements, 4 month in winter and 6 weeks in fall, indicate a fraction of SC related to combustion of non-fossil fuel is in the range 30–50%, which is significantly higher than for EC found in Gothenburg. The results are not directly comparable since the methodology is somewhat different and all measurements represent relatively short campaigns during different parts of the year. However, they still verify that the RWC can be a significant source of BC, even in the city centers. Even though BC, not EC or SC, was calculated in this study, a comparison of the relative contribution of wood combustion could be considered relevant. The results indicate that the annual average fraction of BC from local wood combustion at the Femman station in Gothenburg is 10% of the total BC concentration. For Stockholm the corresponding value at the Torkel Knutssongatan site in central Stockholm is 6%. Since it is likely that also part of the LRT contribution originates from wood combustion, the current study indicates total fractions related to wood combustion above these values.

A compilation of source apportionments for different regions across the world [63] indicates that for urban areas in the north western part of Europe the average fraction of population weighted PM_2.5_ related to domestic burning is 22% and for traffic it is 21%. Corresponding estimates from this study are 20%, 14% and 18% for RWC in Gothenburg, Stockholm and Umea respectively and for local road traffic exhaust the estimates are 10%, 14% and 2%. The significantly smaller contribution from road traffic exhaust in Umea is expected, due to the more rural character of the area. Considering the strong variations found within urban areas (see Figure 4), it is clear that care should be taken when representing whole urban areas with measurements at a single monitoring site. Also, significant variations between different regions in the north western part of Europe can be expected.

Exposure at home addresses as an indicator for personal exposure is a common but rather crude estimate. How exposure at home address relates to the actual exposure depends for example on the mobility of each individual as well as the infiltration of PM into the building [64]. However, there is a lack of relative risk factors from epidemiologic studies based on actual exposure and for this study there was also insufficient information available regarding mobility of the individuals and building properties determining the infiltration of outdoor air. Due to limitations in available data and for consistency with the applied risk factors, ambient exposure at home addresses was considered the best option. Since the relative risks were based on exposure at home addresses, the uncertainty described by the confidence intervals given in Table 3 includes uncertainties related to differences between actual exposure and ambient exposure at homes.

It would be valuable to separate contributions from different sources also in the LRT, thus allowing a more direct comparison to source apportionments based on monitoring data. For future long-term exposure assessments we recommend inclusion of regional modeling on the European scale for this purpose. Using models on larger scale would also make the estimations of LRT less dependent on individual monitoring sites, especially in Gothenburg and Umea, where monitoring data is scarce.

It is noteworthy that even for large Swedish cities the number of premature deaths due to RWC is comparable to those related to road traffic. Thus, a weakness in the present study is the lack of detailed information when describing emissions from RWC in Stockholm and Gothenburg. In order to reduce the uncertainty in the source apportionment, future studies should aim to improve the description of this source category. It is likely that the current trend with improved technology for vehicles, resulting in lower emissions, will continue. At the same time, no significant improvements are expected within the near future for RWC in Sweden. This means that the relative contribution of RWC will increase.

The presented estimates of premature mortality include the combined uncertainties from the exposure estimates at homes as well as assumptions regarding exposure response functions and applied relative risk factors. With this in mind, the number of deaths should preferably be presented as a range with a confidence interval. This requires more information, e.g., regarding the uncertainty of exposure calculations for specific source categories, as well as further research.

## 5. Conclusions

High resolution dispersion models have been used to calculate population exposure to PM_10_, PM_2.5_ and BC during year 2011 for 35 × 35 km^2^ assessment areas centered on three Swedish cities: Gothenburg, Stockholm and Umea. For each source category, population weighted average concentrations have been calculated and relative risk factors have been applied to estimate premature mortality. Different relative risk factors have been applied for PM from local sources and LRT.

For all three cities, the largest part of the exposure to PM_2.5_ and PM_10_ is due to LRT, while for BC the local contribution is slightly larger than the contribution from LRT. This implies that the use of BC exposure as a health indicator increases the focus on local emissions.

The two dominating local sources are road traffic and RWC. Road traffic is the largest local source of PM_10_, while RWC is larger for PM_2.5_. For road traffic both exhaust and wear particles are emitted, with wear particles representing the largest local (mass) contribution for both PM_10_ and PM_2.5_. For exposure to BC, traffic exhaust is the most important source in the two larger cities Stockholm and Gothenburg, while in Umea, RWC is larger.

The exposure contribution from shipping is very small, even in Gothenburg, which is the largest port in Sweden. This is expected since only a small number of people live in the direct vicinity of the harbor or the shipping fairways. The exposure contributions from the source category “other” in Umea and Gothenburg are significant. However, the large uncertainties in the spatial distribution of the emissions of this category should be taken into account while assessing their impact.

Among the local sources, RWC and road traffic cause the most premature deaths. Using PM_2.5_ as an indicator attributes most deaths to RWC, while using BC puts more weight on road traffic exhaust. It is also concluded that fine traffic related particles (<2.5 µm) are likely a more important cause of mortality associated with long-term exposure than the coarser wear related fraction (2.5–10 µm).

The health impact from RWC, together with the uncertainties related to this category, indicates a clear need for improvement of the emission inventories. Considering that the health impact of RWC in parts of Europe may be comparable to that of road traffic, the same level of detail in the description of emissions should be aimed for. This is far from the case today, with few cities having inventories of heating appliances. Also, to allow quantification of uncertainties in the source attribution, more monitoring data in areas dominated by RWC would be needed.

A major finding is that using relative risk factors representing within-city comparisons or using BC as an indicator for PM from local combustion sources, the local sources of PM cause more premature deaths as compared to LRT. This is true for all three cities. When risk assessments are instead based on the total PM_10_ or PM_2.5_ concentrations, and using corresponding relative risk factors based on “between-city” comparisons, LRT is normally attributed a larger impact and there is an evident risk of underestimating the impact of local sources. The results emphasize the importance to resolve within-city gradients in the concentration field when assessing population exposure and raise more concern to reduce local PM and BC emissions in our cities.

## Figures and Tables

**Table 1:** Summary of modeling setups used for the three domains.

	Stockholm	Gothenburg	Umea
**Models**	Airviro Gauss	Airviro Gauss Finite length line source model OML point source model	Airviro Gauss Finite length line source model OML point source model
**Meteorology**	Climatology	hourly time-series	hourly time-series
**Receptor grid**	quadtree from 35 × 35 m^2^ to 500 × 500 m^2^ for traffic, otherwise 500 × 500 m^2^	quadtree from 50 × 50 m^2^ to 3.2 × 3.2 km^2^ for traffic, otherwise 800 × 800 m^2^	quadtree from 50 × 50 m^2^ to 3.2 × 3.2 km^2^ for traffic and RWC, otherwise 800 × 800 m^2^

OML: Operationelle Meteorologiske Luftkvalitetsmodeller (Operational Meteorological Air Quality Models).

**Table 2:** Inhabitants and baseline mortality.

	Ages (Year)	Gothenburg	Stockholm	Umea
**Population**	all	684,127	1,655,490	109,823
	>30	407,206	1,000,890	62,077
**Baseline mortality (per 100,000 inhabitants)**	all	939	751	1009
	>30	1396	1130	1522

**Table 3:** The relative risks used for different sources.

Pollutant	Relative Risk (95% CI) per 10 µg·m^−3^	Reference
Long-range contribution (PM_2.5_)	6% (4–8%)	WHO 2013, Hoek et al. [54]
Local contribution black carbon (BC)	60% (10–110%)	Janssen et al. [18]
Local contribution PM_2.5_	17% (5–30%)	Jerrett et al. [55]
	6% (4–8%)	Hoek et al. [54]
Non-combustion particles (PM_2.5–10_)	1.7% (0.2–3%)	Meister et al. [53]

PM: particulate matter; BC: black carbon; WHO: World Health Organization.

**Table 4:** Percentage of population weighted concentrations related to local sources within the modeling domain.

PM-Fraction	Gothenburg	Stockholm	Umea
PM_10_	28%	27%	20%
PM_2.5−10_	21%	24%	10%
PM_2.5_	36%	30%	27%
BC	70%	55%	57%

**Table 5:** Percentage of premature deaths related to local sources compared to LRT. For premature deaths due to local contributions to concentrations of PM_2.5_ and BC, separate estimates have been made using relative risks from three different studies: Jerrett et al. [55], Hoek et al. [54] and Janssen et al. [18].

Reference	Gothenburg	Stockholm	Umea
Jerrett et al. [55]	77%	70%	69%
Hoek et al. [54]	58%	50%	46%
Janssen et al. [18]	70%	62%	60%

**Figure A2:**
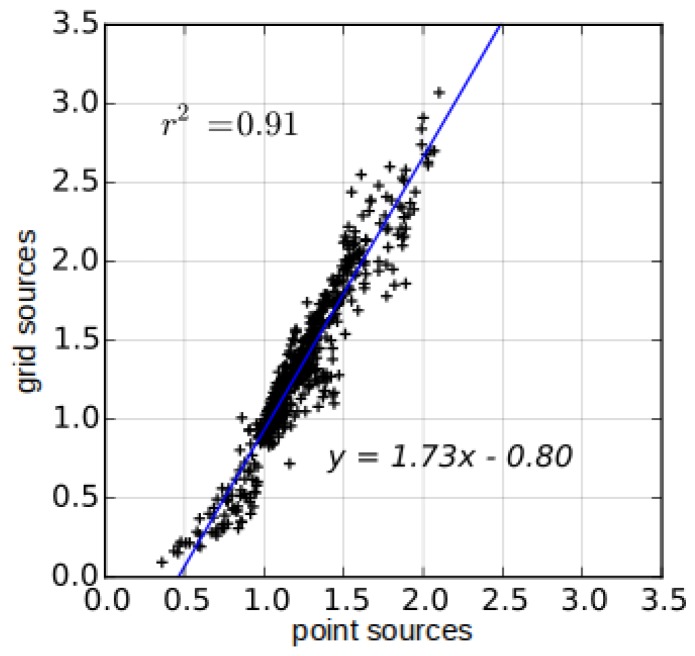
Comparison of different representations of emissions from RWC. The values represent calculated annual average concentrations of PM_2.5_ in µg·m^−3^, related to local RWC at 600 home addresses in Gothenburg, using gridded emissions as well as point-sources.

## Supplementary Materials

The following are available online at www.mdpi.com/1660-4601/14/7/742/s1, S1: gridded emissions with a resolution of 100 × 100 m^2^. GeoTiff raster files are provided for PM_10_, PM_2.5_ and BC, for each assessment area in units of ton/(year·km^−2^). Each band of the raster files corresponds to a source category which is specified in the metadata included in the file. Access to bottom-up emission databases, owned and maintained by air quality administrations of each city, can be granted under a separate agreement mediated by the corresponding author, S2: modelled annual average concentrations with a resolution of 50 × 50 m^2^. GeoTiff raster files are provided for PM_10_, PM_2.5_ and BC, for each assessment area in units of µg·m^−3^. Each band corresponds to a source category, which is specified in the metadata included in the file, S3: Observations used in the evaluation provided as a worksheet together with corresponding model results. The gridded population data used to calculate population weighted concentrations may not be distributed freely, but can be ordered from Statistics Sweden (www.scb.se/en/).

## Appendix B

The consequences of using different ways to represent RWC in the dispersion model, point-sources in Umea and gridded emissions in Gothenburg and Stockholm, were investigated. For the Gothenburg area, a comparison was made with a simulation where the grids were converted to point-sources (one point-source at the center of each grid-cell). The point-sources were given similar properties as in Umea, e.g., a chimney height of 5 m, exhaust gas temperature of 100 °C and parameters related to building downwash. The comparison was made for annual average concentrations of PM_2.5_ related to local RWC at 600 home addresses well distributed over the area. The comparison is presented in Figure A1.

The correlation is high, *r*^2^ = 0.91, which implies that spatial variations are similar in both cases. However, due to inability to reproduce the same release height, including plume lift, the grid sources, as compared to point sources, cause around 25% higher levels for the areas with the highest concentrations and slightly lower concentrations for the areas with the lowest concentrations.

## Appendix C

The comparison between calculated concentrations and measurements is presented in Table A1 for PM_10_, Table A2 for PM_2.5_ and Table A3 for BC. In the tables, “type” refers to the representativeness of the monitoring station, e.g., traffic or UB (Urban Background), “aver mon” refers to annual average of monitoring data, “aver mod” to annual average from model results, “diff (abs)” to absolute difference between annual averages from model and monitoring, “diff (%)” to relative difference between annual averages from model and monitoring and “*n*” to number of samples. IA in the table represents the Index of Agreement (*d*) [65], a standardized measure of the degree of model prediction error which varies between 0 and 1. The Index of Agreement is calculated according to

(A1) $$d=1-\frac{sum\left({\left(obs-sim\right)}^{2}\right)}{sum\left({\left(abs\left(sim-mean\left(obs\right)\right)+abs(obs-mean\left(obs\right)\right)}^{2}\right)}$$

where value of *d* = 1 indicates a perfect match and 0 indicates no agreement at all. It should be noted that if the correlation of yearly variations is low, the IA will be low, even if average values are relatively close.

**Table A1:** Statistics showing the model performance for PM_10_ concentrations.

**City**	Gbg	Gbg	Umea	Umea	Sthlm	Sthlm	Sthlm	Sthlm
**Station**	Haga	Gårda	Bibl.	Västra Esp.	Torkel.	Hornsg.	Sveav.	Norrlandsg.
**Type**	Traffic	Traffic	UB	Traffic	UB	Traffic	Traffic	Traffic
**aver mon**	24.2	25.8	11.4	23.5	14.1	36.5	29.9	29.1
**aver mod**	21.7	27.1	10.2	23.8	13.4	37.6	29.5	33.8
**diff (abs)**	2.5	−1.3	1.2	−0.4	0.7	−1.0	0.4	−4.7
**diff (%)**	11%	−5%	10%	−2%	5%	−3%	1%	−16%
**IA**	0.30	0.09	0.33	0.82	0.94	0.93	0.81	0.80
***n***	5	8	9	6	12	12	10	10

Aver mon: annual average of monitoring data; aver mod: annual average from model results; diff (abs): absolute difference between annual averages from model and monitoring; diff (%): relative difference between annual averages from model and monitoring; IA: Index of Agreement; *n*: number.

**Table A2:** Statistics showing the model performance for PM_2.5_ concentrations.

**City**	Gtbg	Gtbg	Umea	Umea	Sthlm	Sthlm	Sthlm	Sthlm
**Station**	Haga	Gårda	Bibl.	Västra Esp.	Torkel.	Hornsg.	Sveav.	Norrlandsg.
**Type**	Traffic	Traffic	Traffic	Traffic	UB	Traffic	Traffic	Traffic
**aver mon**	11.2	9.4	4.9	7.8	8.1	11.6	9.9	11.5
**aver mod**	8.5	10.6	5.7	11.6	7.7	12.5	11.0	13.0
**diff (abs)**	2.7	−1.2	−0.8	−3.8	0.3	−0.9	−1.1	−1.5
**diff (%)**	24%	−12%	−17%	−49%	4%	−8%	−11%	−13%
**IA**	0.37	0.23	0.57	0.33	0.96	0.95	0.90	0.55
***n***	5	5	6	6	12	12	10	3

**Table A3:** Statistics showing the model performance for BC concentrations.

**City**	Sthlm	Sthlm
**Station**	Torkel.	Hornsg.
**Type**	UB	Traffic
**aver mon**	0.7	3.3
**aver mod**	0.6	2.8
**diff (abs)**	0.1	0.5
**diff (%)**	13%	14%
**IA**	0.00	0.73
***n***	5	5

## Appendix D

**Table A4:** Population weighted annual average concentrations of the different PM-fractions for the different modeling domains presented separately for the different source categories.

Source Category	PM-Fraction	Pop. Weighted Conc. (µg·m^−3^)		
		Gothenburg	Stockholm	Umea
Traffic wear	PM_10_	2.1	2.4	0.34
	PM_2.5–10_	1.7	1.7	0.27
	PM_2.5_	0.41	0.73	0.07
Traffic exhaust	PM_2.5_	0.27	0.21	0.04
	BC	0.23	0.28	0.09
RWC	PM_2.5_	1.3	0.96	0.93
	BC	0.16	0.08	0.12
Other	PM_10_	0.4	0.0	0.4
	PM_2.5–10_	0.06	0.00	0.07
	PM_2.5_	0.33	0.03	0.35
	BC	0.06	0.00	0.06
Shipping	PM_2.5_	0.04	0.02	0.01
	BC	0.01	0.01	0.00
LRT	PM_10_	10.7	9.9	6.8
	PM_2.5–10_	6.5	5.3	3.0
	PM_2.5_	4.2	4.6	3.8
	BC	0.2	0.3	0.2
Sum local sources	PM_10_	4.1	3.6	1.7
	PM_2.5–10_	1.7	1.7	0.3
	PM_2.5_	2.4	1.9	1.4
	BC	0.5	0.4	0.3
Total conc.	PM_10_	15	14	8.5
	PM_2.5–10_	8.2	7.0	3.3
	PM_2.5_	6.5	6.5	5.2
	BC	0.7	0.7	0.5

**Table A5:** Premature deaths estimated for the different modeling domains. Estimates are given separately for the source categories. For local contributions from combustion sources, estimates have been made using relative risks from three different studies: JE: Jerrett et al. [55], HO: Hoek et al. [54], JA: Janssen et al. [18] (see Table 3). RWC: residential wood combustion; LRT: long-range transport.

Source Category	PM-Fraction	Premature deaths								
		Gothenburg			Stockholm			Umea		
LRT	PM_2.5_	72			174			10		
traffic wear, 2.5–10 µm	PM_2.5–10_	18			35			1		
		**JE**	**HO**	**JA**	**JE**	**HO**	**JA**	**JE**	**HO**	**JA**
Traffic, <2.5 µm	PM_2.5_	65	24		177	65		2	1	
	BC			77			184			5
RWC	PM_2.5_	126	47		181	67		15	5	
	BC			53			55			7
Other	PM_2.5_	31	11		6	2		6	2	
	BC			21			1			3
Shipping	PM_2.5_	3	1		4	1		0	0	
	BC			4			4			0
Sum (local sources)		243	101	172	404	171	279	23	9	15
Sum (total)		316	173	245	578	345	452	33	19	26
